# Current Surgical Options for the Management of Pediatric Glaucoma

**DOI:** 10.1155/2013/763735

**Published:** 2013-04-24

**Authors:** Jose Morales, Sami Al Shahwan, Sami Al Odhayb, Ibrahim Al Jadaan, Deepak P. Edward

**Affiliations:** ^1^King Khaled Eye Specialist Hospital, P.O. Box 7191, Riyadh 11462, Saudi Arabia; ^2^The Wilmer Eye Institute, The Johns Hopkins Hospital, 600 N. Wolfe Street, Baltimore, MD 21287, USA

## Abstract

Currently, there are numerous choices for the treatment of pediatric glaucoma depending on the type of glaucoma, the age of the patient, and other particularities of the condition discussed in this review. Traditionally, goniotomy and trabeculotomy ab externo have been the preferred choices of treatment for congenital glaucoma, and a variety of adult procedures adapted to children have been utilized for other types of pediatric glaucoma with variable results and complications. More recently, seton implantations of different types have become more popular to use in children, and newer techniques have become available including visualized cannulation and opening of Schlemm's canal, deep sclerectomy, trabectome, and milder more directed cyclodestructive procedures such as endolaser and transcleral diode laser cyclophotocoagulation. This paper reviews the different surgical techniques currently available, their indications, results, and most common complications to allow the surgeon treating these conditions to make a more informed choice in each particular case. Although the outcome of surgical treatment in pediatric glaucoma has improved significantly, its treatment remains challenging.

## 1. Introduction

Pediatric glaucoma includes a wide variety of conditions which result in elevated intraocular pressure and optic nerve damage, ranging from primary congenital glaucoma since birth to developmental glaucoma associated with other diseases and acquired glaucoma secondary to multiple causes. Depending on the age of the patient, it presents with particular features and circumstances that need to be taken into account and frequently require surgical intervention. Traditional surgical procedures are evolving, and the choices are increasing as diagnostic advances, surgical instrumentation, and newer techniques emerge. The prognosis of the disease has significantly improved over the last half century because of the development of angle surgery, trabeculectomy, seton implantation, and use of antimetabolites [1]. The purpose of this paper is to review the literature on the techniques currently available and their results, for the management pediatric glaucoma, and to offer guidelines on what elements to consider when taking a surgical decision for these patients. 

### 1.1. Background

Several issues, pertinent to the visual outcome of pediatric glaucoma, need to be considered before undertaking surgery. 

*Unique Features of Glaucoma in Infants Are as follows*

- Distensibility of the globe from birth until age 2-3 which can cause stretching at all levels of the eye: cornea (increased corneal diameter), anterior chamber angle (shifting of structures), sclera (globe enlargement and axial myopia, generalized scleral thinning, and localized staphylomas), optic nerve (cupping), scleral canal (enlarged disc diameter), and lamina cribrosa (posterior displacement) [2–7];
- capability to produce amblyopia through persistent media opacities and uncorrected anisometropia or irregular astigmatism [8–10];
- corneal opacities initially caused by epithelial corneal edema and later by permanent stromal edema [10, 11] and breaks in Descemet's membrane.

*Particular Challenges Regarding Glaucoma Surgery in Infants [12] Are as follows*

- Lack of cooperation:
  - requiring examination under sedation or anesthesia before or after surgery;
  - difficulty in protecting consistently and adequately the operated eye, applying medications and monitoring eye for complications and response to surgery.
- Anatomic differences: smaller palpebral fissure and less rigid and thinner sclera, different than expected location of landmarks in buphthalmic eyes.
- First surgery has a greater chance of success. This is important because it needs to last longer than in adults because of longer life expectancy. Therefore, it is not advisable to be performed by the occasional or untrained surgeon on this kind of surgery. It has been recommended by some that it should be done at an ophthalmic referral center that receives at least 20 new cases per year as well as having considerable experience with adult glaucoma surgery [13]. Considerable volume and experience of the referral center in dealing not only with skillful glaucoma surgery but also with safe anesthesia is very important [1].

In order to review in a more systematic way the surgical approaches to the wide variety of glaucomas in children, we will refer to the different glaucomas in children as follows [1, 14]:

- primary congenital glaucoma (newborn and infantile), when an isolated idiopathic developmental abnormality of the anterior chamber angle exists.
- Glaucomas associated with congenital anomalies; aqueous outflow is reduced due to congenital ocular or systemic disorder.
- Acquired glaucoma; the outflow impairment is the result of acquired ocular disease or systemic abnormality.

Histopathological correlation has been attempted to support this classification with Group I considered mainly affecting the trabecular meshwork (trabeculodysgenesis), while Group II is thought to result from abnormalities in trabecular meshwork, iris, and anterior segment (iridotrabeculodysgenesis) [15, 16]. This histopathogenic classification has been proposed as the basis to decide whether to perform angle surgery versus a filtering procedure. A problem with this approach is that although a predominance of histopathological abnormalities may exist in some cases, there are also many instances where a combination of elements exists and the decision is still taken based on clinical manifestations, age of appearance, and severity of the disease. 

## 2. Primary Congenital Glaucoma

The traditional staged approach outlined in most textbooks or review papers on primary congenital glaucoma involves what has been denominated as “angle surgery” (goniotomy in cases with relatively clear corneas) and trabeculotomy (for those with cloudier cornea). If the initial procedure fails then it can be repeated, usually more than twice with goniotomies and twice with trabeculotomies. The next usual procedure is either standard trabeculectomy with the use of antimetabolites or combination of trabeculotomy/trabeculectomy. If this fails, the typical next procedure (in an eye which still has a reasonable visual potential) would be a tube implantation. And finally when everything else has failed or the visual potential is so poor, a cyclodestructive procedure which can be repeated several times for IOP or pain control is recommended [17]. 

### 2.1. Angle Surgery as First Procedure

For patients with the following features:

1. no other ocular or systemic abnormalities,
2. disease noted at least one month after birth but before one year of age, and
3. with corneal diameters less than 14 mm.

“Angle surgery” typically consists of either goniotomy or trabeculotomy ab externo [17]. Goniotomy (if the cornea is clear enough for adequate visualization of the goniotomy knife passing across the anterior chamber and incising the trabecular meshwork) or trabeculotomy ab externo if the cornea is cloudy enough to preclude a safe goniotomy (or when the surgeon prefers this technique due to prior training or more experience with the procedure even when the cornea is clear). Both procedures presumably work by allowing a more direct access of aqueous humor into Schlemm's canal and the outflow system. 

#### 2.1.1. Goniotomy

This is the oldest procedure described for treating congenital glaucoma. Although initially used by De Vincentis, in 1893, for all types of glaucoma [18], Barkan is credited with combining it with gonioscopic view, giving a detailed description of the procedure and reporting its successful use in congenital glaucoma in 1938 [19]. Modifications allowed to combine goniotomy with the use of the surgical microscopes, which were introduced into ophthalmology in the early 1950's [20, 21], but routinely used in large eye institutions dealing with this disease (such as Moorfields Eye Hospital in London) until the late 1960's [22]. 

The main aims and steps of the procedure have remained unchanged and include entering the anterior chamber through a clear corneal incision and crossing the anterior chamber to the opposite side to incise the trabecular meshwork (while visualizing the angle with a surgical gonio lens) and covering an arc of 100–110 degrees [23]. 

The results are excellent when used in patients fulfilling the criteria outlined above. In 1953, Barkan himself described his 17-year results of treating congenital glaucoma with goniotomy and reported an 80% success rate in 188 eyes, with adequate pressure control without medications [24]. Shaffer described 287 operated eyes and stated that one or two goniotomies cured 94% of patients diagnosed with glaucoma between 1 month and 24 months of age [25]. Broughton and Parks also reported their 20-year experience with 50 eyes of patients with primary congenital glaucoma who underwent goniotomy, obtaining an overall 88% success rate (with a mean followup of five years) after one or more goniotomies [26]. 

However, Shaffer's 94% outstanding success rate dropped to 30% when glaucoma was present at birth or after the age of two years [25]. It was suggested that the type of angle abnormality was responsible for the level of success obtained and that this procedure was particularly suited for those cases with incomplete separation of the iris from the trabecular meshwork, which prevented the necessary separation of the corneoscleral trabecular sheets [23]. 

Goniotomy represented an important breakthrough in ophthalmology because for the first time it was possible to treat congenital glaucoma in a successfully and reasonably safe way. The pioneering work of Dr. Barkan and others, with gonioscopy to perform successful goniotomies, opened new roads in the understanding, diagnosis and treatment of glaucoma in general. 

**Figure 1:**
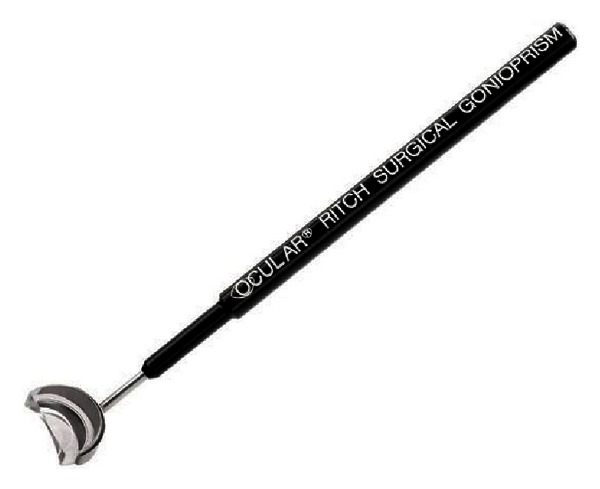
Ritch direct panoramic gonioscopy lens.

Useful adjuvants for the procedure such as the use of viscoelastic material [27–29] to allow for a safer pass of the goniotomy knife across the anterior chamber and the use of newer goniolenses that can be utilized with the current surgical microscopes have improved the safety margin and adequate visualization for the goniotomy knife application. A wide variety of direct gonioscopy lenses have been used including the Koeppe, Barkan, and Swan Jacob lens. More recently, other useful modifications have been introduced such as the Ritch direct panoramic gonioscopy lens, which gives a 160 degree view, obstructing only half the cornea and leaving plenty of working space. (Ritch R, Personal communication; Ocular Instruments Inc.; Bellevue, Washington) (Figure 1).

Initial results of goniotomy in primary congenital glaucoma have been excellent in western populations and with the characteristics outlined above (93.5% of eyes controlled at one year), but longer followup studies demonstrate that risk of relapse is a problem, even 30 years after the initial procedure [22]. They noted that patients with symptoms at birth and those who required more than one goniotomy were more likely to relapse. 

A recent study from East Africa [30] reported on the results of goniotomy in 36 children, with advanced disease and late presentation, followed for an average of 1 year. The study suggested that almost 40% of those followed for more than 3 months required repeat goniotomy for adequate control. Only 8.5% of corneas were clear preoperatively, while 78% were clear postoperatively. 

 In Saudi Arabia, however, a study including 254 goniotomies had a success rate of only 52% with the worse outcomes in moderate and severe glaucomas (only 13% and 0% success rates, resp.) [31]. These poor results with primary goniotomy (and also with trabeculotomy) prompted this institution to start utilizing combined trabeculotomy/trabeculectomy plus mitomycin C as a primary procedure, especially in those more severe cases of primary congenital glaucoma or glaucomas associated with other congenital abnormalities which in that study happened to be the majority (70%) of patients [32]. 

Ideally, the pupil should be constricted, before starting the procedure, to minimize the risk of damaging the lens when crossing the anterior chamber and to facilitate the retraction of the iris from the angle. This can be accomplished with preoperative pilocarpine drops or with intracameral miotic agents. 

Some preliminary studies with a small number of patients suggest the possibility of using endoscopic goniotomy to treat cases of congenital glaucoma where a cloudy cornea would preclude this type of procedure [33, 34].

#### 2.1.2. Trabeculotomy

Trabeculotomy was described in 1960 by Burian who unroofed Schlemm's canal through an incision radial to the limbus and entered it with a specially made instrument that he called trabeculotome. He called the procedure trabeculotomy ab externo in contrast to goniotomy which was considered a trabeculotomy ab interno [35]. Later, in 1966, Harms modified the technique by dissecting a superficial scleral flap similar to the one used in trabeculectomy and then making the radial incision to identify Schlemm's canal and opening it with a modified instrument (Harm's trabeculotome) which had two parallel arms, one to open Schlemm's canal and the other one to guide externally the direction of the trabeculotome [36]. Pilocarpine drops and/or intracameral miotic agents are advisable to constrict the pupil before the procedure for the same reasons cited for goniotomy. Pilocarpine 1% drops three times daily to the operated eye for two to three weeks after the operation have also been recommended to contract the ciliary muscle holding it away from the trabeculum during the healing phase [37].

This technique became fairly popular and preferred by some [38] over goniotomy for the treatment of congenital glaucoma. Controversy regarding which was a better initial procedure (trabeculotomy versus goniotomy) existed since the introduction of trabeculotomy and remained for a long time, with some authors arguing that trabeculotomy had a better success rate as a single procedure than goniotomy [39]. 

When the results of both procedures were compared in several studies [40–42], the conclusion was that they were similar and satisfactory for both techniques, and the argument more or less was settled. Quigley reported observations based on 28 trabeculotomies, performed at the Wilmer Eye Institute, with adequate control of IOP and stable or improved optic disc status in 80% of eyes followed for one year or longer. Main complication was anterior chamber hemorrhage which was mostly self-limited and did not require intervention [37]. Less common reported complications are iridodialysis, ruptured Descemet's membrane, and staphyloma formation [43]. There is general consensus that trabeculotomy is the preferred procedure for mild primary congenital glaucoma when corneal opacity would preclude the performance of a goniotomy. There are advantages and disadvantages for each procedure outlined in table I but the final decision to perform goniotomy versus trabeculotomy (as an initial procedure in primary congenital glaucoma) rests on the surgeon and includes his/her personal preference and familiarity with the technique (Table 1). 

### 2.2. Subsequent Procedures When Angle Surgery Fails at Least Twice or as Primary Procedures When Angle Surgery Is Not the Procedure Most Likely to Succeed

#### 2.2.1. Trabeculectomy

Trabeculectomy has the advantage of being an operation more familiar to most ophthalmologists, and it has been advocated by some as a primary procedure in congenital glaucoma [44–46]. Its mechanism of action is bypassing the aqueous from the anterior chamber to a subconjunctival fistula. However, trabeculectomy presents special difficulties in childhood glaucoma. The eye is large and the limbal anatomy is frequently distorted, and lack of familiarity with these unusual eyes can lead to complications such as iris and ciliary body incarceration and vitreous loss [44, 47–50]. Another problem is that the superior conjunctival area is utilized, and even after successful surgery, with a normal life expectancy, there is a high likelihood that a significant proportion of these patients will need further surgery within their lifetimes [47]. Eyes with a previous failed trabeculectomy have a higher failure rate in the long term. Use of antimetabolites has been associated with complications such as flat anterior chambers, hypotony, choroidal detachments, and endophthalmitis in patients with congenital glaucoma and therefore should be reserved for patients with more severe glaucomas or that have failed initial angle surgery [47, 51]. Milder antimetabolite agents (5-FU) have been also suggested for patients with risk factors for failure [47]. 

The age at presentation has been identified as a risk factor for failure when performing primary “angle surgery”. Of 50 eyes in which infantile glaucoma was diagnosed at birth, only 26% had pressure controlled by one or two goniotomies. The other 74% (37 eyes) required multiple goniotomies, trabeculotomies, trabeculectomies, and cyclocryotherapy with some of them never reaching control [25]. Similar poor results were obtained in those diagnosed as late developing infantile glaucoma after the age of 24 months [2]. Severity and duration of glaucoma are other negative predictive factors for success with either surgery [52]. In such cases, with anticipated worse prognosis, a primary trabeculectomy with antimetabolites [53] has been performed by some. 

A study at Wills Eye Hospital showed a clear preference for trabeculectomy in primary congenital glaucoma as a secondary procedure when angle surgery failed, while it was used mostly as a primary procedure in aphakic glaucoma patients [54]. Results at one year were abysmal in aphakic patients (0% success) compared with 76.9% in phakic patients, even when combined with mitomycin C. Other studies in patients with primary congenital glaucoma and secondary phakic glaucomas also reported reasonable results with mitomycin C trabeculectomy [51, 55]. Concern about long-term complications in pediatric patients with a long life ahead of them has been raised because the thin, avascular bleb observed sometimes with this kind of surgery can produce late bleb leaks, bleb-related endophthalmitis, and long-term hypotony [56–61]. There is also certain reluctance to utilize mitomycin and other antimetabolites in children [62] because of the possibility, at least in theory, of secondary neoplasms as it occurs in other areas of pediatrics. Because trabeculectomy without mitomycin C in children, who have failed other glaucoma procedures, has a much lower and unacceptable success rate (less than 50% at 18 months in a retrospective study) [48], enhancement with these agents is widely utilized. 

Mitomycin C trabeculectomy in pediatric patients results in reasonable success rates of 67–87% at one year; however, a significant drop to 58-59% at two years has been reported by two different authors [61, 63], reflecting the higher tendency in children compared to adults to scar and occlude the filtering site [64]. 

Besides the use of antimetabolites, other modifications that have been suggested to improve the outcome of this surgery in pediatric patients have been the use of a fornix base flap because of its lower rates of bleb-related infections [65], use of releasable sutures, and use of Healon GV left into the anterior chamber at the end of the procedure to prevent early postoperative hypotony and shallow anterior chamber [66]. In these very elongated eyes, Luntz and others have recommended to perform the sclera-corneal incision at the most anterior location possible, since a corneal site entry reduces the risk of blocking the opening by the ciliary body and iris adherences as well as vitreous loss [49, 67]. Unlike adults, where paracentesis tends to be self-sealing, in young children it can leak profusely due to increased tissue elasticity. In our institution, we routinely close the paracentesis tract at the end of the procedure with an absorbable suture (10–0 polyglycolic acid suture with spatulated needle) and bury the knot. If this suture becomes loose before it reabsorbs, it is important to remove it to avoid the risk of suture-related microbial keratitis. Cycloplegic agents are useful in the immediate postoperative period to relax the ciliary body and facilitate deepening of the anterior chamber. 

#### 2.2.2. Combination Trabeculotomy Ab Externo and Trabeculectomy

Maul et al. first reported the use of this procedure in 1980, for a child with severe bilateral primary congenital glaucoma who had not been controlled with initial goniotomy [68]. After that, the results of this procedure have been mostly reported in Middle Eastern and Indian populations, where it was used preferentially because it was felt that congenital glaucoma did not respond as well to angle surgery as previously described in western populations [31, 69, 70]. The intended mechanism of action for combining these two procedures is to gain access to dual outflow, through Schlemm's canal and/or the trabeculectomy fistula. One study in Saudi Arabia which prompted an institution to search for other options, such as this combined procedure, reported a disappointing 67% success rate with trabeculotomy alone at one year follow-up. [50]. They hypothesized that the poor results compared with those from the western literature were because these patients had more severe degree of disease due to a higher rate of consanguinity and poor prognostic indicators such as larger corneal diameters, presentation since birth, and higher intraocular pressures. 

A small study of nine Arab children, with primary congenital glaucoma, who underwent primary trabeculotomy/trabeculectomy before one year of age, demonstrated a much better success rate of 93.5% in contrast with 72% of those that underwent trabeculectomy alone [69]. A theoretical advantage of the combined procedure is that it provides two major outflow pathways, the incised trabecular meshwork by the trabeculotome and the excised trabecular meshwork block and filtering bleb by the trabeculectomy [32, 69]. A practical advantage was that in those cases where Schlemm's canal could not be identified, something that has been reported in up to 11–15% of trabeculotomies [37, 69], the procedure would not result in failure, since the trabeculectomy pathway could still function [69]. 

Mandal et al. reported a 94.4% success rate of primary trabeculotomy-trabeculectomy in 122 eyes with primary congenital glaucoma operated in India, with a mean follow-up of approximately a year. Complications included one case each of premature penetration into anterior chamber, vitreous prolapse and Descemet detachment, plus 10 cases of shallow anterior chamber from which only two required surgical reformation [70]. 

Al-Hazmi et al., at King Khaled Eye Specialist Hospital a tertiary level referral center in Saudi Arabia, studied retrospectively a very large sample of 532 patients (820 eyes), who underwent goniotomy, trabeculotomy, or combined trabeculotomy-trabeculectomy with mitomycin C as an initial glaucoma procedure [31]. Almost 70% of eyes had either moderate or severe glaucoma as per corneal enlargement (>13 mm) corneal haze and higher levels of IOP. They found a clear correlation between success rate and severity of the disease. All procedures resulted in high success rates (>80%) for the mild form of primary congenital glaucoma. However, combined trabeculotomy-trabeculectomy with mitomycin C gave the best results for moderate (80%) and severe (70%) cases of primary congenital glaucoma in contrast with only 40% and 10%, respectively, for trabeculotomy in those cases with moderate and severe glaucoma. 

An important point to make regarding the combined procedure is that although it has been described by several authors as making the trabeculectomy (incision and scleral block excision) an extension of the initial incision for the trabeculotomy [68–70], it can be done using an alternate technique. Our preference is to make a separate incision [32], limbal and more anterior, right under the hinge of the scleral flap, when entering the anterior chamber and then to perform a sclerotomy using controlled bites with the Kelly Descemet punch. This maneuver minimizes the chances of iris and ciliary body prolapse and incarceration mentioned by some authors [47, 49]. 

#### 2.2.3. Glaucoma Drainage Implants (GDIs)

Glaucoma drainage implant surgery has a definitive role in managing infants and other children with glaucoma refractory to angle surgery and trabeculectomy. A tube is placed in the anterior chamber of the eye and aqueous flows through the tube and into the subconjunctival space to a plate which is placed at least 8-9 mm posterior to the limbus. 

The first glaucoma drainage implant used in the pediatric population was the Molteno implant (IOP Inc., Costa Mesa, CA, USA) in 1973 [71], followed by the Baerveldt implant (Pharmacia and Upjohn Inc., Kalamazoo, MI, USA) [72, 73] and Ahmed valve implant (New World Medical Inc., Rancho Cucamonga, CA, USA) [74]. The Ahmed valve implant has a unidirectional valve restriction flow mechanism, designed to open when the aqueous pressure is higher than 8 mmHg [75]. This is highly effective in reducing the risk of early postoperative hypotony compared to nonvalved implants (Molteno, Baerveldt), which require special surgical maneuvers to reduce the flow or a two-stage procedure to avoid this problem. 

Molteno, Baerveldt, and Ahmed implants have been the most common devices used in children and at the present time, judging from the current literature [76], Baerveldt and Ahmed implants are those mostly used (the choice depending mostly on surgeon preference and individual case circumstances). 

Comparison of GDIs (Ahmed and Baerveldt) versus MMC trabeculectomy in children younger than 2 years of age in a retrospective, age-matched, comparative study resulted in better IOP control with the GDIs than the MMC trabeculectomy group with cumulative success rates of 87% versus 36%, respectively, at one year and even a larger difference of 53% versus 19% at 6 years [77]. Complication rates requiring reoperation though were more frequent among the GDIs patients than among those with mitomycin-C (MMC) trabeculectomy (45.7% versus 12.5%, resp.). A prospective, randomized study comparing Ahmed implant versus MMC trabeculectomy in pediatric aphakic glaucoma [78] seemed to show higher qualified success in the Ahmed (67%) versus the MMC trabeculectomy group (40%) and complication rates higher in the MMC trabeculectomy (40%) than Ahmed implant (26.7%) although the differences were not statistically significant. 

Success figures for individual aqueous shunt devices vary widely (31–93%) [79], but it is difficult to compare the success rates because different studies include very different populations, lengths of follow-up, surgical techniques, and types of devices [72, 79]. For instance, after two years of follow-up, one study found a very low success rate of 31% [80] while another one had 86% success at the same interval [81]. However, the first study evaluated much younger patients, operated within the first two years of life, while the latter looked at older patients (average age 6 years). 

Molteno, Baerveldt, Shocket, Krupin, Ahmed, and Optimed implants have been all used in children [82]. Currently, Baerveldt and Ahmed implants are the most commonly used in adults and children (the choice depending mostly on surgeon preference and individual case circumstances). 

Several studies with Ahmed implants in the pediatric population have suggested that congenital glaucoma may be associated with higher failure rate than other pediatric glaucomas [83–85]. Other studies, however, did not find a correlation between surgical failure and glaucoma type in the pediatric population [81, 86, 87]. 

One of the largest studies and with longer follow-ups of GDIs (Ahmed and Baerveldt) in pediatric glaucoma [76] included 38 eyes with congenital glaucoma and 32 eyes with aphakic glaucoma. One-year success rates were 92% and 90% in the congenital and aphakic groups, respectively, but decreased to 42% and 55% after 10 years. There was a preference to implant Ahmed valve in congenital glaucoma while Baerveldt was preferred for aphakic patients. Another study [87] of Ahmed implant in children reported a high cumulative success rate of 89% at 6 months just after two years, and they explained that their lower longer-term success than other studies might have been caused because of their use of smaller plate sizes in a number of patients. 

Age of the patient did not clearly affect success rates of GDIs [81, 88], but complications and rate of re-operations seem to be higher than in adults [79]. Some authors utilizing AVG did not find a correlation between failure and prior glaucoma surgery [81, 87], while others [84, 89, 90] noted that eyes with previous glaucoma surgeries showed significantly worse results. 

There is a limited and contradictory information with respect to the effect of intraoperative adjunctive MMC use and GDIs in pediatric glaucoma. Several studies of aqueous shunt implantation with and without adjunctive MMC in adults did not show a benefit to intraoperative MMC use [91–93], and it had been assumed that the same was true for pediatric patients. Several authors [82, 94] confirmed no post operative difference, in IOP measurement or complications, with or without MMC in studies including children. But then, Al-Mobarak and Khan [80] surprisingly found a shorter survival time (22 months versus 16 months) and a much lower cumulative probability of success (31% versus 80%), respectively, when comparing the patients treated with Ahmed and mitomycin C and those without it. It has been argued that because of the retrospective nature of this study, a selection bias could have occurred with the mitomycin C patients having worse type of glaucomas [79, 95, 96]. Because of the lack of evidence that mitomycin C improves the outcomes with GDIs and because of the potential complications, it is generally agreed that it is better to avoid its use [79, 97]. 

The size of the implant is another important consideration because it has been demonstrated that the degree of IOP reduction achieved postoperatively is directly proportional to the end plate size. This correlation is observed up to a certain degree, since a study comparing Baerveldt 500 mm^2^ versus 350 mm^2^ did not show lower intraocular pressures with the larger implant [98]. Currently the Ahmed implant comes in a smaller “pediatric” version denominated FP8 model with 96 mm^2^ surface (9.6 mm wide/10 mm long) and “adult” version or FP 7 model with a larger surface of 184 mm^2^ (13 mm wide/16 mm long). In our experience, it is almost always possible (except for nanophthalmic and premature baby eyes) to place the larger adult size Ahmed implant in children, and this should increase the chances of obtaining lower intraocular pressures than with the smaller implant size. 

**Figure 2:**
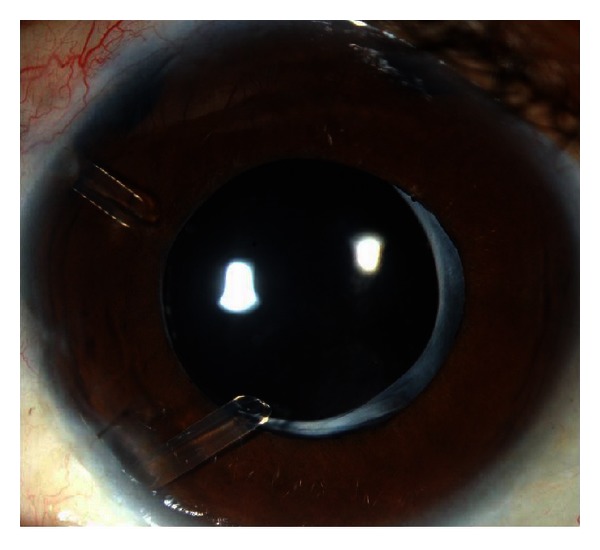
Eye from patient with pediatric glaucoma with two glaucoma drainage implants.

Studies in adults have shown encouraging results after the use of a second implant when the first one had failed [99–101]. Surgical management of a child with an already failed aqueous shunt with a second shunt implantation in a different quadrant, without removing the first one, is possible. A couple of studies in children which utilized the AGV reported reasonable rates of success after the second implant [87, 88]. Ou et al, treating primary congenital glaucoma, reported a cumulative probability of success of 86% at 1 and two years and 69% at five years [88] after the second AGV implant (Figure 2). 

Postoperative complications after GDIs in children are numerous, some of them occurring more frequently than in adults [79] and requiring postoperative intervention more often [102]. This higher incidence of complications in children compared to adults is probably related to different factors such as buphthalmic eyes with a thinner sclera, a growing orbit and eye, and more frequent eye rubbing in children. Use of Ahmed implant has been described in special glaucomas that require minimizing hypotony as much as possible, such as Sturge-Weber syndrome, and the results have been satisfactory [103]. With nonvalved implants such as Baerveldt or Molteno, temporary occlusion of the tube with different modalities (internal occlusion with a removable suture, external ligation with reabsorbable sutures or, a combination of both) have been utilized to minimize immediate postoperative hypotony. 

Those complications that are either more commonly cited or more serious, endangering the eye or vision, that need to be taken into consideration are as follows.

**Figure 3:**
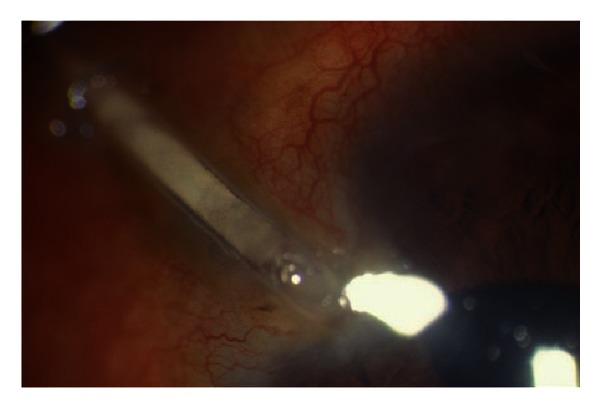
Eye of patient with extruded tube.

1. Early postoperative complications (within one week after surgery): shallow [77, 84, 104] and flat anterior chamber [78, 88], hypotony, hyphema [84], choroidal detachment [82, 84] and suprachoroidal hemorrhage [78, 83], corneal tube contact [77, 86, 88], cataract formation [77, 84], secondary pupil and iris abnormalities [73, 76], and retinal detachment [87].
2. Intermediate postoperative period (after one week to three months) hypertensive phase [85, 87, 105], hypotony after suture removal [106], Bleb encapsulation [107].
3. Late complications (three months to years): tube exposure [88] (Figure 3), endophthalmitis which is often associated with tube extrusion [81, 108], fibrous ingrowth [77], cyclitic membrane and persistent hypotony [73], and ocular motility abnormalities [74, 84, 109–111]. Adult studies have attempted to determine whether either one of the most utilized GDIs (Ahmed and Baerveldt) is more successful and/or safer than the other one [112], and the results so far have been mixed without giving clear superiority to one device over the other, except for perhaps a slightly lower IOP reduction for Baerveldt and fewer and less serious complications for Ahmed. For children, we do not have prospective, randomized studies to evaluate this issue, and therefore the choice of the specific device is determined by surgeon personal experience, preference, and availability of the shunt and special circumstances of the case. Either one offers advantages or disadvantages that need to be taken into account, especially for these more complicated patients. Preferences around the Baerveldt implant cite a lower incidence of encapsulation and lower intraocular pressures with less medications, while the Ahmed implant is preferred because of less immediate postoperative hypotony complications prevented by the valve mechanism.

#### 2.2.4. Cyclodestructive Procedures

Cyclodestructive Procedures in pediatric glaucoma are usually reserved for those challenging cases that have failed multiple more conservative treatments and for those patients with anatomic abnormalities that preclude traditional surgeries. [113]. Their mechanism of action is through ablation of the ciliary body and resultant reduction of aqueous production. 

*Cyclocryotherapy.* This procedure was introduced since 1950 [114] and decreased the intraocular pressure by freezing and destroying the ciliary body epithelium [115]. Its use as a primary procedure in congenital glaucoma produced poor results [116]. Other devastating complications including phthisis, retinal detachment, and sympathetic ophthalmia have been reported [117–119]. A long-term evaluation study in pediatric patients reported a higher incidence of phthisis bulbi in aniridic patients [120]. Although cyclocryotherapy was utilized for a while for refractory and poor visual potential pediatric patients [121], it is not a preferred cyclodestructive procedure any longer because it has been gradually replaced by less aggressive and more targeted procedures such as laser cyclophotocoagulation either transclerally [122, 123] or endoscopically [124, 125], which result in less inflammation and complications [126]. 

*Transcleral Diode Laser Cyclophotocoagulation.* Introduced in the early 1990's [127], it rapidly replaced other laser methods of transcleral cyclophotocoagulation [128, 129] used before. Transcleral YAG laser cyclophotocoagulation in children resulted in very disappointing results, with one study reporting that after ten patients were treated, only half had controlled IOP, and there was loss of vision in four patients and phthisis bulbi in one [130]. Transcleral diode laser cyclophotocoagulation, on the other hand, had a convenient compact design and less side effects, in particular avoiding the occurrence of sympathetic ophthalmia, a dreaded complication of YAG transcleral cyclophotocoagulation [131, 132]. 

Diode laser cyclophotocoagulation was utilized in a variety of pediatric glaucomas since its introduction, but the reports suggested that the response in children was less than in adults [133, 134]. It was hypothesized that younger eyes may recover faster from the treatment than older patients. 

An overall success rate of 50% in pediatric refractory glaucomas has been cited, including retreated patients (average 2.2 procedures per eye) and a high retreatment rate of 70% and most failures occurring during the first 6 months after treatment [113]. 

**Figure 4:**
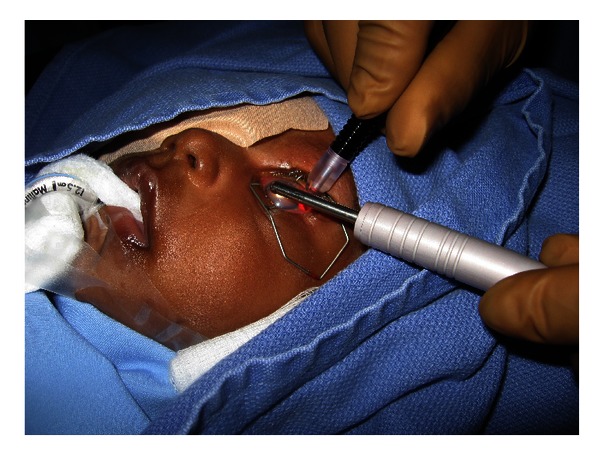
Transcleral diode laser cyclophotocoagulation being performed in a child with congenital glaucoma refractive to other treatments. Transillumination probe being utilized to aid in the correct localization of ciliary body.

A problem with performing adequate transcleral cyclophotocoagulation in congenital glaucoma eyes with aberrant anatomy features is to get accurate localization of the ciliary body. Transillumination, which we routinely use at our institution with a fiber optic probe, is recommended [113, 135].  Figure 4. After the procedure, there is no significant decrease in the number of glaucoma medications and it is considered mostly as an adjunctive therapy [113, 134]. 

Recurrence of elevated intraocular pressure is common [134, 135]. A relatively large study of 77 pediatric glaucoma eyes [134] noted an initial adequate IOP reduction in 62%, but this fell to 37% by one year. Repeat treatments in 72% accomplished useful IOP reduction for a year or more, but 13% of patients did not respond at all. Noted complications were retinal detachment in three eyes and significant inflammation in 10% of eyes. No significant reduction in number of medications was observed. 

Diode laser transcleral cyclophotocoagulation still should be reserved for patients with limited visual potential (20/100 or worse), while other incisional procedures should be attempted for patients with better visual acuity [113]. 

Reasonable indications for this procedure are (1) advanced glaucoma with previous failed multiple procedures; (2) markedly elevated IOP on acute presentation, where at least temporary IOP control is required before undertaking more definitive surgery; (3) treatment of a blind painful eye with an elevated IOP; (4) markedly elevated IOP, where the fellow eye has undergone surgery and it is desirable to defer surgery until the fellow eye is more stable; (5) moderately elevated IOP with maximum medical therapy where the risks of drainage surgery are high (severe complications in the fellow eye) or where incisional surgery was declined by parents [113]. 

Some authors suggest caution when cycloablation surgery fails and tube surgery is undertaken (to consider two-stage tube procedure), since it has been noted that some cases are associated with chronic postoperative hypotony [86, 106]. 

**Figure 5:**
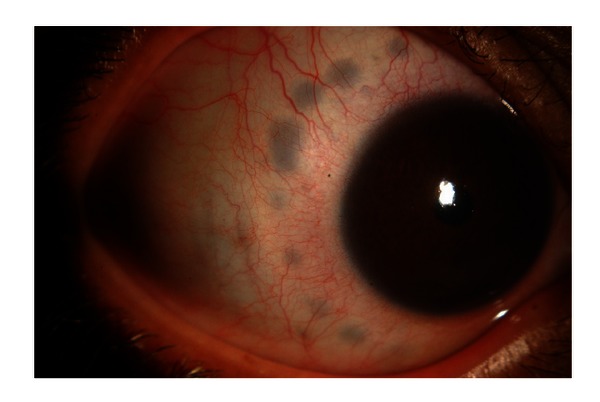
Scleral thinning spots after transcleral diode laser cyclophotocoagulation in pediatric patient.

A complication observed with diode laser cyclophotocoagulation, predominantly in younger patients, is scleral thinning [136] (Figure 5). It is probably an overlooked complication because in many cases it tends to be mild and without clinical implications. Although probably very rare, actual scleral perforation requiring suturing of the sclera has been reported [137, 138], and this highlights the need to lower the energy levels utilized for pediatric glaucoma cases with thinner sclera. In our institution, we tend to start at roughly half the level of initial energy utilized for adults and then increase gradually until a mild “popping sound” is heard, which is the attempted threshold. 

*Endolaser Cyclophotocoagulation (ECP).* Described first in 1992 [139], this procedure accomplishes cycloablation through direct visualization (in contrast with other transcleral cycloablation procedures which just estimate the location of the ciliary body). It uses a 20 gauge instrument, with endoscopic view through a monitor and a diode laser treating each individual ciliary process until whitening and shrinkage is observed. 

Initial use in children was tentative [124], due to the concern of possible serious complications or phthisis bulbi from this new procedure. It was utilized first in a few eyes for a pilot study, with poor visual prognosis and with limited amount of cyclophotocoagulation (180 degrees). After it was felt that it was relatively safe, it was used in patients with better visual potential, with higher levels of energy, and a wider extent of treatment (270 degrees). The results were encouraging with no sight-threatening complications, severe hypotony, or significant pain or inflammation. At 3 years of follow-up, 50% (five eyes) were considered success and 50% (five eyes) failures. 

A larger study by the same group [125], including 36 eyes that were followed for an average period of a year and a half, described their wider experience. Patients were treated for 180–270 degrees. Their success rate was 34% with one quarter of eyes needed retreatment at least once. Cumulative success rate after all procedures was 43%. Postoperative complications included retinal detachment in 2 patients, prephthisis in 1 patient, and progression of vision loss from hand motion to no light perception in 1 patient. All complications occurred in aphakic patients. They concluded that this procedure is moderately effective for the management of difficult pediatric glaucomas and that an aphakic patient may have an increased risk of complications. It is worth noting that a number of phakic patients (14) were treated in this study and they did not find any new development of cataracts after the procedure. 

Endolaser cyclophotocoagulation offers the advantage of more targeted, visualized end point of treatment and ability to titrate the amount and extent of treatment as advantages, while adding the potential possible complications associated with intraocular procedures (infection, suprachoroidal serous and hemorrhagic detachments, and intraocular pressure spikes related to viscoelastic retention). In our opinion, it is the most useful resource in pseudophakic and aphakic patients who are not candidates for some reason for tubes or that have thin and abnormal scleras that would prevent the use of transcleral cyclodestructive procedures. Although no significant complications have been reported from its limited use in phakic patients, we do not recommend its use in these patients because of the high risk of damage to the crystalline lens during the procedure.

*360 Degree Trabeculotomy*. The rational for performing this kind of surgery is that a larger extent of exposed Schlemm's canal will yield a lower intraocular pressure than partial opening of the lumen. 360-degree trabeculotomy surgery was initiated by Smith in 1960 [140] in cadaver eyes using suture material. He described using two separate radial incisions to thread a piece of nylon into Schlemm's canal and tensioning the suture from both ends opening into the anterior chamber. Beck and Lynch in 1995 [141] refined the technique by using 6–0 polypropylene suture, which was threaded all around the 360 circumference of Schlemm's canal and reported success of 87% of treated eyes with congenital glaucoma. In 2011, Beck et al. [142] reported a 77% success of same procedure in cases of primary congenital glaucoma considered to have a poor prognosis (onset at birth, presentation after 1 year of age, failure of initial goniotomy). 

Visual outcomes and intraocular pressure (IOP) control have been shown to be better with 360-degree trabeculotomy than with multiple goniotomy procedures [143]. However, when threading Schlemm's canal with a suture, there is a risk of misdirection into the suprachoroidal space [144, 145]. Use of an illuminated microcatheter avoids this potential complication as the tip with the illuminated (flashing or steady light) continuously indicates its position within the canal or whether it starts to go astray [146]. 

Girkin et al. recently [146] reviewed the results of 11 eyes with primary or secondary congenital or juvenile glaucoma that underwent circumferential trabeculotomy performed with an illuminated microcatheter and reported a 91.6% qualified and 83.3% unqualified success rate with short-term (8 to 12 months) followup. Transient hyphema was common, but no major complications were seen in this series. 

In another retrospective consecutive chart review of 16 eyes [147], there was a 47.0% reduction in IOP at 6 months, although average antiglaucoma medications use was not significantly reduced from baseline. 

The superiority of canaloplasty over other more traditional techniques in adults still needs to be demonstrated by randomized, controlled studies that utilize only one intervention (instead of several interventions added to the basic procedure of Schlemm's canal catheterization such as deep sclerectomy, circumferential vasodilatation of the canal, tensioning of the canal with a nylon ligature). Still we believe that 360-degree trabeculotomy with a lighted probe offers significant advantages over the traditional trabeculotomy technique in children, and it deserves further consideration. In contrast with traditional trabeculotomy. In contrast with traditional trabeculotomy, this procedure adds certainty with regards to adequate identification and probing of Schlemm's canal for the whole360-degree circumference in a single session. 

Potential risks are that while retrieving the more rigid (than the prolene suture) catheter throughout the anterior chamber damage to the lens or other structures, a more extensive Descemet detachment than that produced by a traditional trabeculotome could occur. 

**Figure 6:**
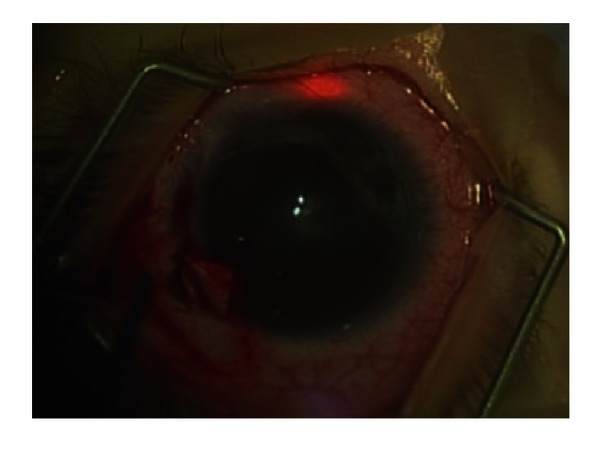
360 degree trabeculotomy in a newborn child. Illuminated catheter progressing around Schlemm's canal at 12 o'clock evidenced by red light.

We have utilized this procedure for a limited number of congenital glaucoma cases, with both the 250 micron catheter (iScience Interventional, MenloPark, CA) or a battery operated smaller caliber catheter with apparently reasonable preliminary results (Unpublished data) (Figure 6). We believe that a prospective, randomized, controlled study comparing this procedure with either trabeculotomy or goniotomy in congenital glaucoma patients with similar level of pathology is necessary to make a more definitive conclusion about its place in the treatment of this kind of glaucoma. 

*Deep Sclerectomy*. Nonpenetrating surgery has attracted more interest during the last decade [148–150] for its potential to decrease intraocular pressure without some of the immediate postoperative hypotony and long-term bleb complications of traditional filtering surgery. Its use in pediatric glaucoma has been fairly circumscribed, and there are only a handful of studies describing its results in the English ophthalmic literature [149, 151]. 

**Figure 7:**
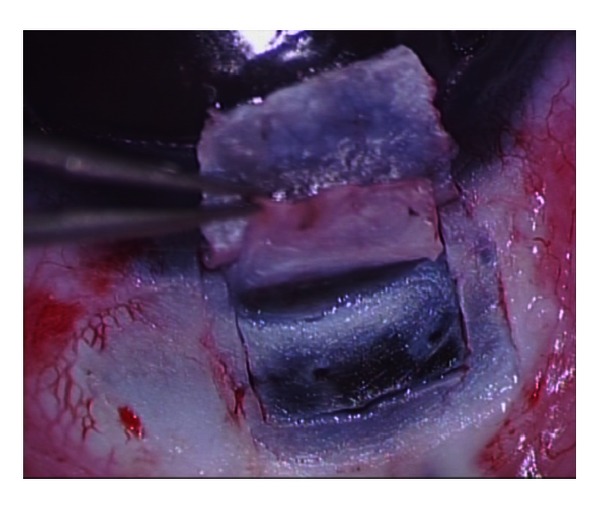
Deep sclerectomy performed in a a child with glaucoma secondary to Sturge-Weber Syndrome.

Deep sclerectomy involves the dissection of a deep scleral flap, deroofing of Schlemm's canal, and preserving the structural integrity of the trabecular meshwork [152]. Its mechanism of action is not entirely clear, but a combination of a more diffuse filtering bleb formation and uveoscleral and transcleral flow have been cited [153]. It has been proposed by some [154, 155] as an alternative to other procedures in high risk pediatric glaucoma cases such as Sturge-Weber syndrome, where it is desirable to minimize sudden hypotony and the resultant possibility of massive choroidal serous or hemorrhagic detachments, which can lead to catastrophic outcomes. (Figure 7). 

Its use has been reported in a few studies for the treatment of primary congenital glaucoma [156] and congenital glaucoma refractory to treatment [151]. It has been proposed as an alternative to nonangle surgery, because it potentially reduces the complications of immediate postoperative hypotony and overfiltration and the side effects of performing a peripheral iridectomy and avoids the complications of long-term filtering blebs, including serious intraocular infections. Although its risk profile appears better than penetrating procedures such as trabeculectomy, its utilization has not become so generalized partly because it is technically more demanding and because it is technically more demanding and also because of surgeon wide variability in fashioning the deep scleral flap [152, 157]. 

Prospective, randomized, comparative studies with other traditional procedures are still lacking and are difficult to perform, because pediatric glaucoma is an uncommon disease in most places and matching of study samples is more difficult with the variety and levels of disease on these patients. 

Because in Saudi Arabia congenital glaucoma is more common and a more severe disease than in other countries [31, 50], dealing with the severe problems and complications of traditional penetrating surgery has prompted trying alternatives, such as deep sclerectomy. Preliminary results from an ongoing study reviewing the results of deep sclerectomy, as a primary procedure, in 74 eyes with primary congenital glaucoma and at least three years of follow-up suggest an overall success rate of 82.4% [158]. No catastrophic complications were seen. 

Difficulties with this procedure in children are that they have a thinner and more elastic sclera and variable anatomical features, and it is not always possible to identify Schlemm's canal [156], all of which make a procedure already technically demanding, even more challenging and more likely to be performed at highly specialized centers in treating this condition. 

One study [151] reported abysmal results (100% failure) of the procedure in eight patients who had already failed other glaucoma surgeries and a high rate of failure to successfully perform the procedure as well as serious complications including a case of vitreous hemorrhage and other with vitreous loss and retinal detachment. Other authors have reported much better results when they used deep sclerectomy as an initial procedure in congenital glaucoma, with a success rate of 75% at last follow-up [156] and therefore advocate this technique as a primary intervention. 

Most recently Feusier et al. [149], one of the main advocates of deep sclerectomy, published the results of performing combined deep sclerectomy and trabeculectomy in 35 eyes of patients with a variety of pediatric glaucomas with a mean follow-up of almost four years. They reported a complete and qualified success rates, based on cumulative survival curves, after 9 years of 52.3% and 70.6%. Failures were more common among more severe cases as expected. 

Useful pearls when performing deep sclerectomy in pediatric glaucoma are (1) more careful dissection of the deep flap; (2) if antimetabolites are used, to apply them before dissecting the superficial scleral flap; (3) not to attempt deroofing of Schlemm's canal as this tissue is difficult to identify and peel and may result in perforation. In our experience, deep sclerectomy, when done properly, is another helpful and relatively safe procedure in the armamentarium to manage congenital glaucoma especially in its mild form. Further data with a relatively larger number of patients should be available to the ophthalmic community in the near future. We do not advocate the use of this procedure for cases with other congenital secondary glaucomas, where the disease process may be more complicated and the angle may be closed or abnormal. We hope that its use may also contribute to a better understanding of congenital glaucoma mechanisms. 

*Trabectome*. Ab interno trabeculectomy using a mechanical device such as the trabectome has been mainly used for adult forms of glaucoma [159]. Its aim and presumed mechanism of action is to enhance outflow via increased access to Schlemm's canal, allowing aqueous to escape the anterior chamber without the impedance of the strip of trabecular meshwork and inner wall of Schlemm's canal that are removed. In order to perform this procedure in an efficient manner, the cornea needs to be relatively clear in order to clearly visualize the anterior chamber angle, and trabecular meshwork landmarks must be clearly visible. When the anterior chamber is deep and the angle structures clearly visualized in pediatric glaucoma with a large cornea, this procedure is suitable. However, corneal clouding and/or presence of Haab's striae may preclude clear visualization of the anterior chamber angle. In addition, aberrant or incomplete development of the meshwork and Schlemm's canal complex in pediatric glaucoma [160] may prevent electrocautery stripping of the meshwork. Therefore, one could envision the trabectome being potentially useful in milder cases of primary congenital glaucoma and other forms of pediatric glaucoma, where the angle structures are well developed and presence of Schlemm's canal identified. It may also be useful in other secondary forms of glaucoma where the angle remains relatively open such as what may be seen in pediatric glaucoma associated with certain forms of uveitis. 

There are no case series that specifically address the use of trabectome in pediatric glaucoma. The use of the trabectome in pediatric glaucoma was described by Minckler and colleagues in a large case series presented at the American Ophthalmological Society along with the published discussion. However, specifics of the technique, types of patients, and outcomes were not clearly described in this paper [159]. Future studies focusing on this specific minimally invasive surgical technique will hopefully provide new information on the benefit of the trabectome in pediatric glaucoma. 

*Goals of the Surgery*. Although for every glaucoma procedure the most immediate objective is the reduction of harmful elevated intraocular pressure, in children other aims need to be kept in mind when deciding which procedure to perform and when to do it. With children we are running against time because the sooner the child develops clear media, improved visual acuity, and binocularity the better. It is well known that in spite of adequate normalization of IOP, many elements hamper the development of a normal vision in these children; however, aiming at preserving or restoring the best possible visual function is an important final goal. 

While choosing a procedure it is important to keep in mind that the immediate goals of the surgery are normalization of the intraocular pressure and clearing of the cornea as soon as possible. In the long term, the aims of the procedure are prevention of (further) optic nerve damage and peripheral vision integrity and preservation or restoration of the capability to develop as close as normal binocular visual function. 

## 3. Conclusions

The management of pediatric glaucoma in its different forms is still quite challenging and the visual and long-term results variable depending on the severity and type of disease. The number and type of newer surgical procedures and modifications to traditional ones have improved our choices and capability to treat this condition. Although it is generally agreed that angle surgery is the best initial approach for milder cases of primary congenital glaucoma, the surgical procedure to use for more severe cases, secondary glaucomas, or failed angle surgery cases is less clear cut. Prospective, randomized, comparative studies are scarce because of the infrequent and variable nature of the condition, but the surgeon facing such patients still needs to make the best informed choice regarding which procedure to use and what are the chances of success. Some procedures, like goniotomy, except for some minor modifications, are almost in their eighties but have stood the test of time and remained as useful and strong as when they revolutionized the field of congenital glaucoma surgery at first. Others like trabeculotomy are in their fifties and also have maintained their status in the initial management of congenital glaucoma, keeping the same indications. Others, like trabeculectomy, also in their fifties have undergone a number of important modifications, such as the use of antimetabolites to improve the outcome and the shift to fornix base conjunctival opening to encourage more diffuse, posterior, thicker blebs, but even then still have a significant rate of failure and complications especially in pediatric patients. There are some procedures in their 20's like the Baerveldt and Ahmed implants which have made a big difference in the management of difficult or refractory cases that before would have been candidates only for cyclodestructive procedures, which also have become more refined and targeted allowing using them at earlier stages. And finally, the newest procedures (360° trabeculotomy, trabectome, and deep sclerectomy) barely getting to their first decade still need to prove themselves in a disease that requires long-term, life-long control. In the end, the continued efforts of many researchers, surgeons, and clinicians on this field have improved the outlook and chances for the life of our young patients with such difficult disease which has life-lasting consequences. 

## Figures and Tables

**Table 1:** Summary of the advantages and disadvantages for either procedure.

Goniotomy		Trabeculotomy	
Pros	Cons	Pros	Cons
Simpler	Requires a degree of corneal clarity	It can be done even with cloudy cornea	More elaborate procedure (it requires accurate identification of Schlemm's canal)

Faster procedure	Introduction of sharper instruments across the anterior chamber (higher risk of damage to intraocular structures), and an assistant is needed to tilt the patient's head	No need to introduce sharp instruments across anterior chamber	More lengthy procedure

Does not disturb conjunctiva	Better success before age 3	It can be converted to trabeculectomy	It disturbs conjunctiva

Direct visualization of TM		Eliminates entire TM as obstacle and works in situations with multiple mechanisms exist, such as impermeability of inner wall or collapse of Schlemm's canal	

More targeted cutting of abnormal tissue in primary congenital glaucoma		Success reported even in patients older than 3	

May repeat one or more times			May repeat only one time

If the cornea is sufficiently clear it is usually possible to identify target tissue		Not always able to find Schlemm's canal (3–15% cases)	
